# Nursing Students’ Perceptions, Challenges, and Barriers in Applying the Nursing Process During Clinical Training in Cambodia: A Cross‐Sectional Study

**DOI:** 10.1155/nrp/6401733

**Published:** 2026-05-30

**Authors:** Sokha Yem, Lida Vann, Leapchakriya Loem, Sokunthea Kem, Sreypeov Tun, Nov Sreyroth

**Affiliations:** ^1^ Faculty of Nursing, University of Puthisastra, Phnom Penh, Cambodia, puthisastra.edu.kh

**Keywords:** Cambodia, clinical competency, clinical training, family-centered care, flipped classroom, nursing education, nursing process, nursing students, situated learning, theory-practice gap

## Abstract

**Background:**

The nursing process is a systematic framework for delivering safe, individualized patient care and is embedded within nursing curricula in Cambodia. Nonetheless, empirical evidence examining nursing students’ experiences when applying this framework during clinical training remains scarce.

**Objective:**

To examine nursing students’ perceptions of the nursing process and to identify the practical challenges and organizational barriers encountered during clinical practice at a private university in Phnom Penh, Cambodia.

**Methods:**

A cross‐sectional online survey was conducted among 170 nursing students with confirmed clinical experience enrolled in ADN and BSN programs at the University of Puthisastra. A structured self‐report questionnaire assessed perceptions (12 items), challenges (10 items), and barriers (10 items). The instrument demonstrated strong content validity (S‐CVI/Ave = 0.93) and acceptable internal consistency (Cronbach’s α = 0.82–0.88).

**Results:**

Participants reported positive perceptions (mean = 3.93 ± 0.68), with 81.6% agreeing that the nursing process facilitates better clinical decisions. Moderate challenges were reported (mean = 3.32 ± 0.68), principally patients’ inability to provide sufficient assessment information (56.1%), documentation variability across hospitals (50.9%), and difficulty obtaining consent for physical assessment (48.6%). Perceived barriers were lower (mean = 2.79 ± 0.91), with heavy workload (30.2%), insufficient background knowledge (28.8%), and limited role modeling (28.3%) most frequently identified. BSN students reported significantly higher perceptions than ADN students (*p* = 0.014); Year 4 students reported significantly fewer barriers than Year 2 students (*p* = 0.027).

**Conclusion:**

Nursing students hold favorable views of the nursing process but face substantive practical and organizational constraints during clinical training. These constraints are mainly related to patient assessment limitations, variability in documentation systems, workload pressures, and limited role modeling and clinical support. The findings highlight the need for standardized documentation and strengthened clinical mentorship, as well as improved collaboration between academic institutions and clinical settings. They also suggest that simulation‐based learning, flipped classroom approaches, technology‐enhanced education, and family‐centered communication training may be considered as potential strategies in future curriculum development to address the identified gaps. Further multisite research, including public and provincial institutions, is recommended to enhance generalizability.

## 1. Introduction

The nursing process is a systematic, evidence‐based framework that guides nurses in delivering safe, individualized, and holistic patient care [1]. Comprising five interconnected steps—assessment, diagnosis, planning, implementation, and evaluation—it provides a structured approach for clinical reasoning and decision‐making in nursing practice [2]. It is widely recognized as a core professional competency and is embedded in nursing curricula globally [3].

### 1.1. The Cambodian Context

Cambodia’s healthcare system continues to develop following decades of reconstruction. The Ministry of Health’s Core Competency Framework for Nurses emphasizes systematic assessment, individualized care planning, and evaluation as essential competencies [4]. Nursing education has expanded across diploma, associate degree, and bachelor programs in both public and private institutions [5], with the nursing process integrated into curricula.

However, the system continues to face constraints common in low‐ and middle‐income countries, including limited resources, high patient loads, variable supervision, and inconsistent clinical standards across facilities [6]. These factors may affect students’ ability to consistently apply classroom knowledge in clinical practice.

### 1.2. Theory–Practice Gap and Educational Context

International evidence consistently reports a theory–practice gap in nursing education, where students experience difficulty applying theoretical knowledge in clinical settings [7, 8]. Studies from multiple low‐resource settings show that implementation of the nursing process is often hindered by workload, limited supervision, documentation challenges, and insufficient learning support [9–12].

Although various educational approaches such as simulation, blended learning, and student‐centered teaching have been discussed in the literature, these are context‐dependent and have not been systematically evaluated within Cambodian nursing education settings.

### 1.3. Theoretical Framework

This study is grounded in situated learning theory, which emphasizes that learning occurs through participation in real clinical environments where students gradually develop competence through guided practice [13]. From this perspective, the extent to which students can apply the nursing process depends not only on knowledge acquisition but also on the learning conditions within clinical practice environments.

### 1.4. Research Gap and Study Purpose

While previous studies in Cambodia have explored challenges faced by practicing nurses and students in clinical settings [14–16], there is still limited quantitative evidence focusing specifically on nursing students’ perceptions of the nursing process together with the challenges and organizational barriers they experience during clinical training.

This gap is important because understanding students’ perspectives is essential for informing curriculum development and improving alignment between classroom learning and clinical practice environments.

Accordingly, this study aimed to

1. examine nursing students’ perceptions of the nursing process,
2. identify challenges encountered during its application in clinical training,
3. explore perceived barriers in clinical settings, and
4. compare differences across program types and academic years.

## 2. Methods

### 2.1. Study Design and Setting

A cross‐sectional survey design was employed at the University of Puthisastra, a private institution in Phnom Penh that offers ADN (3‐year) and BSN (4‐year) nursing programs and maintains clinical partnerships with multiple public and private hospitals.

### 2.2. Participants and Sampling

The target population comprised ADN and BSN nursing students who had completed at least one clinical rotation of two or more weeks. One clinical week in this study refers to five working days (Monday to Friday), with approximately 6–8 h of clinical practice per day depending on hospital placement schedules.

A nonprobability convenience sampling technique was used. All eligible students were invited to participate, and those who responded voluntarily were included in the study.

Of 237 eligible students, 212 completed the survey; 170 confirmed clinical experience, yielding an effective response rate of 71.7%. This sample size exceeded the minimum of 150 required to detect a medium effect size (*d* = 0.50) with 80% power at α = 0.05 [9, 17].

### 2.3. Instrument Development and Validation

#### 2.3.1. Adaptation and Content Validity

The questionnaire was adapted from validated instruments by Koy et al. [17] and Areri et al. [9], with permission. It comprised four sections: demographics (8 items), perceptions (12 items), challenges (10 items), and barriers (10 items), rated on a 5‐point Likert scale.

Each item was scored from 1 (Strongly Disagree) to 5 (Strongly Agree). Higher mean scores indicated more positive perceptions, greater challenges, or stronger perceived barriers depending on the construct. No reverse‐coded items were included.

Three nursing education experts evaluated each item for relevance, clarity, and simplicity. The scale‐level content validity index (S‐CVI/Ave) for the three domains was 0.91, 0.95, and 0.93, respectively, indicating excellent content validity [18]. Five items with I‐CVI < 0.67 were revised based on expert feedback. Five items with I‐CVI < 0.67 were revised based on expert feedback; detailed examples of expert comments and corresponding item revisions are presented in Supporting Table S1. The instrument was pilot tested with 20 students, resulting in minor wording refinements.

Internal consistency in the final sample was acceptable to good for all domains: perceptions (α = 0.88), challenges (α = 0.85), and barriers (α = 0.82).

### 2.4. Data Collection

Data were collected from March to April 2024 via Google Forms distributed through institutional email and learning management systems. Electronic informed consent was obtained prior to access to the survey. Completion required approximately 10 min; one reminder was sent after two weeks.

### 2.5. Data Analysis

Data were analyzed with SPSS Version 27.0 (α = 0.05). Descriptive statistics summarized demographic characteristics and item responses; responses of 4 or 5 were combined as overall agreement. Independent‐samples *t*‐tests compared ADN and BSN students; one‐way ANOVA with Tukey post hoc tests examined differences across academic years; Pearson correlations examined relationships among the three constructs. Assumptions of normality and homogeneity of variance were verified prior to all parametric tests.

### 2.6. Ethical Considerations

The study received approval from the University of Puthisastra Research Ethics Committee (Approval No. 020UPRC, February 15, 2024). Participation was voluntary and anonymous; electronic informed consent was obtained before survey access.

## 3. Results

### 3.1. Participant Characteristics

Table 1 presents demographic data. Mean age was 22.01 ± 1.87 years; 69.4% were aged 21–23 years; 78.8% were female. BSN students comprised 54.7%; ADN students, 45.3%. By year, 23.5% were in year 2, 44.1% in year 3%, and 32.4% in year 4. Participants had completed a mean of 3.2 ± 1.4 clinical rotations (cumulative experience: mean = 8.7 ± 4.3 weeks). This corresponds to approximately 43–70 clinical days of supervised clinical exposure, based on a clinical week defined as five working days (Monday–Friday), with 6–8 h per day depending on hospital placement. Most trained in tertiary hospitals (86.5%). Self‐rated clinical competence was 3.41 ± 0.73 on a 5‐point scale.

**TABLE 1:** Demographic characteristics of participants (*N* = 170).

Characteristics	*n* (%) or mean ± SD
Age (years), mean ± SD	22.01 ± 1.87
18–20	32 (18.8)
21–23	118 (69.4)
24+	20 (11.8)
Gender	
Female	134 (78.8)
Male	34 (20.0)
Prefer not to say	2 (1.2)
Program	
BSN	93 (54.7)
ADN	77 (45.3)
Year of study	
Year 2	40 (23.5)
Year 3	75 (44.1)
Year 4	55 (32.4)
Clinical rotations (mean ± SD)	3.2 ± 1.4
Total clinical experience, weeks (mean ± SD)	8.7 ± 4.3
Clinical training sites (multiple)	
Tertiary hospitals	147 (86.5)
Provincial hospitals	58 (34.1)
Health centers	21 (12.4)
Self‐rated clinical competence (1–5)	3.41 ± 0.73

Abbreviations: ADN, Associate Degree in Nursing; BSN, Bachelor of Science in Nursing.

### 3.2. Perceptions of the Nursing Process

Participants held strongly favorable perceptions (mean = 3.93 ± 0.68, α = 0.88) (Table 2). The most endorsed items were that the nursing process helps make better clinical decisions (81.6%), followed by its intention to use it as a registered nurse (80.2%), its ability to organize patient care (79.7%), and its ability to improve communication with the healthcare team (77.4%). The lowest‐rated item was understanding each step clearly (mean = 3.78; 66.5% agreement).

**TABLE 2:** Students′ perceptions of the nursing process (*N* = 170).

Item	Mean ± SD	Agreement *n* (%)
The nursing process helps make better clinical decisions	4.16 ± 0.75	139 (81.8)
I intend to use the nursing process as a registered nurse	4.11 ± 0.78	136 (80.0)
The nursing process helps organize patient care	4.07 ± 0.81	135 (79.4)
The nursing process improves communication with the healthcare team	4.02 ± 0.79	131 (77.1)
I believe it can be applied in all healthcare settings	4.00 ± 0.82	131 (77.1)
It helps give care that fits each patient’s needs	4.00 ± 0.86	128 (75.3)
It helps me evaluate whether my care is effective	3.97 ± 0.85	128 (75.3)
Using the nursing process helps me think critically	3.89 ± 0.85	127 (74.7)
I learned about it in both class and clinical training	3.87 ± 0.86	124 (72.9)
I feel confident using it in clinical practice	3.80 ± 0.86	119 (70.0)
The training I received is sufficient to use the nursing process	3.79 ± 0.81	115 (67.6)
I understand each step of the nursing process clearly	3.78 ± 0.82	113 (66.5)
Overall perceptions	3.93 ± 0.68	

*Note:* Agreement = Agree (4) or Strongly Agree (5); Cronbach’s α = 0.88.

BSN students (mean = 4.02 ± 0.64) reported significantly higher perceptions than ADN students (mean = 3.81 ± 0.70), t(168) = 2.50, *p* = 0.014. Year 4 students (mean = 4.08 ± 0.63) reported higher perceptions than year 2 students (mean = 3.75 ± 0.72), F(2, 167) = 4.23, *p* = 0.016.

### 3.3. Challenges in Applying the Nursing Process

Students reported moderate challenges (mean = 3.32 ± 0.68, α = 0.85) (Table 3). The most frequent was patients’ inability to provide sufficient assessment information (56.1%), followed by documentation variability across hospitals (50.9%) and difficulty obtaining consent for physical assessment (48.6%). Approximately 37%–43% reported difficulty understanding assessment forms, limited knowledge for accurate nursing diagnoses, difficulty communicating with patients or families, difficulty accessing patient data, overly complex forms, trouble obtaining information from team members, and uncertainty about data collection for certain conditions. No significant differences were found between program types (*p* = 0.498) or academic years (*p* = 0.164).

**TABLE 3:** Students′ challenges in applying the nursing process (*N* = 170).

Item	Mean ± SD	Agreement *n* (%)
Some patients cannot give enough information for the assessment	3.57 ± 0.88	95 (55.9)
Different hospitals use different nursing process forms	3.33 ± 0.96	86 (50.6)
I sometimes find it hard to get consent for a physical assessment	3.41 ± 0.79	83 (48.8)
Some questions on assessment forms are hard to understand	3.30 ± 0.94	74 (43.5)
I have limited knowledge to make accurate nursing diagnoses	3.32 ± 0.85	73 (42.9)
I find it difficult to communicate with some patients or families	3.25 ± 0.92	71 (41.8)
It is difficult to access patient data (e.g., labs, medications)	3.30 ± 0.90	70 (41.2)
Nursing process forms are too long or complicated	3.31 ± 0.86	67 (39.4)
I have trouble getting information from other team members	3.23 ± 0.92	66 (38.8)
I am unsure what data to collect for certain conditions	3.18 ± 0.90	62 (36.5)
Overall challenges	3.32 ± 0.68	

*Note:* Agreement = Agree (4) or Strongly Agree (5); Cronbach’s α = 0.85.

### 3.4. Barriers to Applying the Nursing Process

Perceived barriers were comparatively lower (mean = 2.79 ± 0.91, α = 0.82) (Table 4). The most frequently endorsed barrier was heavy workload (30.2%), followed by insufficient background knowledge (28.8%) and rarely observing role models using the nursing process (28.3%). Approximately one‐quarter of students identified insufficient training, inadequate institutional support, lack of time, insufficient mentoring, and limited support from medical staff. Low motivation (24.1%) and lack of confidence (20.8%) were the least reported. Year 4 students reported significantly fewer barriers (mean = 2.58 ± 0.85) than year 2 students (mean = 3.04 ± 0.98), F(2, 167) = 3.68, *p* = 0.027. No significant difference was found between program types (*p* = 0.296).

**TABLE 4:** Students′ barriers to applying the nursing process (*N* = 170).

Item	Mean ± SD	Agreement *n* (%)
The workload in clinical placements makes it difficult to apply	2.92 ± 1.09	51 (30.0)
I do not have enough background knowledge	2.85 ± 1.08	49 (28.8)
I rarely see role models using the nursing process	2.84 ± 1.07	48 (28.2)
I have not received enough training to use it well	2.78 ± 1.05	46 (27.1)
Hospitals do not provide enough support or encouragement	2.78 ± 1.07	45 (26.5)
I do not have enough time during clinical shifts	2.78 ± 1.00	44 (25.9)
There are not enough instructors or mentors to guide me	2.78 ± 1.05	43 (25.3)
Some doctors or staff do not support the nursing process	2.75 ± 1.11	43 (25.3)
I feel unmotivated to use the nursing process during clinical practice	2.74 ± 1.05	41 (24.1)
I do not feel confident using the nursing process	2.73 ± 1.04	35 (20.6)
Overall barriers	2.79 ± 0.91	

*Note:* Agreement = Agree (4) or Strongly Agree (5); Cronbach’s α = 0.82.

### 3.5. Summary of Scale Scores

A summary of scale scores, including mean values and internal consistency, is presented in Table 5.

**TABLE 5:** Summary of scale scores (*N* = 170).

Scale	Items	Range	Mean ± SD	Cronbach’s *α*
Perceptions	12	1–5	3.93 ± 0.68	0.88
Challenges	10	1–5	3.32 ± 0.68	0.85
Barriers	10	1–5	2.79 ± 0.91	0.82

### 3.6. Correlations Between Constructs

Pearson correlations revealed significant relationships (Table 6). Perceptions were negatively correlated with barriers (*r* = −0.31, *p* < 0.001), indicating students with more positive views perceived fewer barriers. Challenges and barriers were positively correlated (*r* = 0.42, *p* < 0.001). Perceptions and challenges showed a weak negative correlation (*r* = −0.16, *p* = 0.039).

**TABLE 6:** Correlations between study variables (*N* = 170).

Variable	1. Perceptions	2. Challenges	3. Barriers
1. Perceptions	1.00		
2. Challenges	−0.16^∗^	1.00	
3. Barriers	−0.31^∗∗∗^	0.42^∗∗∗^	1.00

^∗^ *p* < 0.05.

^∗∗∗^ *p* < 0.001.

## 4. Discussion

This study examined nursing students’ perceptions, challenges, and barriers related to the nursing process at a private university in Phnom Penh, Cambodia. Findings reveal favorable attitudes toward the nursing process coexisting with moderate practical challenges and meaningful organizational barriers that constrain consistent clinical application—a pattern consistent with the theory–practice gap documented internationally [7, 8]. Importantly, this discussion extends the theoretical and educational implications of these findings by integrating evidence on flipped learning, technology‐enhanced education, and family‐centered care (FCC) as promising approaches to address the identified gaps.

### 4.1. Positive Perceptions Despite Implementation Difficulties

Students’ strong positive perceptions (mean = 3.93) reflect recognition of the nursing process as a foundational framework for organizing care, supporting clinical decision‐making, and enhancing interprofessional communication—consistent with studies from Ethiopia, Turkey, Pakistan, Japan, Namibia, and Kenya [8–12, 19]. The persistent gap between favorable attitudes (mean = 3.93) and moderate implementation challenges (mean = 3.32) mirrors globally documented theory–practice divides.

From a situated learning perspective [13], this disconnects signals that clinical learning environments may fail to provide adequate opportunities for legitimate peripheral participation. Without institutional structures—standardized documentation, consistent role modeling, and protected learning time—students cannot fully integrate theoretical knowledge into professional practice despite sound conceptual understanding.

The significantly higher perceptions reported by BSN versus ADN students (*p* = 0.014) likely reflect differences in theoretical depth and clinical exposure between the two programs. Flipped classroom strategies, shown by Özbay and Çınar [20] to significantly improve nursing students’ engagement and knowledge acquisition, could help elevate perceptions among ADN students by enabling more active, student‐centered engagement with nursing process content—particularly through preclass video lectures, interactive case vignettes, and in‐class simulation—before clinical placement. Year 4 students’ higher perceptions relative to year 2 counterparts (*p* = 0.015) are consistent with situated learning theory’s emphasis on competency development through accumulated authentic practice.

### 4.2. Assessment, Communication, and FCC Challenges

The most prevalent challenge—patients’ inability to provide sufficient assessment information (56.1%)—underscores communication and health literacy barriers characteristic of Cambodian healthcare contexts. Cambodia’s population encompasses diverse ethnic minorities, varying educational backgrounds, and traditional health beliefs that shape patient–provider interactions [5]. Language barriers, limited health literacy, and cultural norms around information disclosure complicate comprehensive nursing assessment.

Difficulty obtaining consent for physical assessment (48.6%) and communicating with patients or families (42.0%) further exemplify these challenges. In Cambodia’s collectivist cultural context, family involvement in healthcare decisions is deeply normative, requiring students to navigate complex family dynamics while respecting patient autonomy. The integration of FCC principles into nursing curricula represents a particularly well‐evidenced response to these challenges. Çınar Özbay et al. [21] demonstrated that embedding FCC within a nursing course via distance education significantly enhanced students’ holistic assessment competencies and their capacity to engage families as active care partners—competencies with direct applicability to Cambodia’s cultural landscape. These findings strongly indicate that FCC‐oriented nursing process instruction, culturally adapted assessment training, health literacy strategies, and structured communication workshops should be integrated into Cambodian nursing curricula to address the assessment and interaction challenges identified in this study.

### 4.3. Documentation Variability as a Systemic Structural Challenge

Half of students (50.9%) reported that hospitals employ different nursing process documentation forms—a significant structural inconsistency in Cambodia’s healthcare system that contrasts sharply with high‐income countries, where standardized electronic health records and nursing terminologies (e.g., NANDA‐I, NIC, NOC) increasingly create consistent documentation environments. For students rotating across multiple facilities, this variability imposes cognitive burden, reduces learning efficiency, and prevents competency consolidation. Similar documentation challenges have been documented in Ethiopian and Iraqi contexts [9, 22].

Technology‐enhanced education offers a promising pathway to mitigate this challenge: digital nursing process templates, e‐documentation platforms, and mobile decision‐support applications accessible across clinical sites could standardize students’ documentation experiences and reinforce consistent nursing process habits regardless of facility‐specific forms [16]. Addressing this systemic problem ultimately requires coordinated action among educational institutions, hospital administrators, the Cambodian Nurses Association, and the Ministry of Health to establish national standards for nursing process documentation.

### 4.4. Knowledge Gaps in Nursing Diagnosis: Flipped Learning as a Solution

Students’ difficulty in making accurate nursing diagnoses (42.9%) and uncertainty about data collection for certain conditions (36.8%) reflect challenges in knowledge application. Nursing diagnosis is the most conceptually demanding step of the nursing process, requiring integration of pathophysiology, clinical assessment data, and clinical reasoning to identify human responses to health problems [19]. In Cambodia, this difficulty is compounded by limited resources for Khmer‐language nursing diagnoses, clinical environments that prioritize task completion over independent nursing judgment, and high patient acuity and turnover in tertiary hospitals.

Situated learning theory suggests that such knowledge–practice gaps may be better addressed through learning approaches that integrate clinical reasoning with authentic or simulated learning experiences. In the literature, flipped classroom approaches have been reported as one potential strategy to support nursing students’ preparation for clinical reasoning by enabling preclass engagement with theoretical content followed by in‐class application activities [20]. However, these approaches were not examined in the current study.

Additional strategies such as concept mapping, case‐based learning, and problem‐based learning have also been reported in previous studies as supportive methods for developing diagnostic reasoning skills [23, 24]. These should be interpreted as contextually relevant educational approaches suggested in the literature rather than interventions evaluated in this study.

### 4.5. Workload, Time Constraints, and Technology‐Enhanced Learning

Heavy workload (30.2%) and insufficient clinical time (25.9%) emerged as primary organizational barriers. Cambodia’s healthcare system faces critical nursing shortages, with ratios well below international recommendations [6]. In tertiary hospitals where most students train, nurses commonly manage 15–20 or more patients per shift, with minimal time for comprehensive assessment, care planning, and documentation. When students observe staff skipping nursing process steps due to time pressure, they may internalize task‐focused rather than process‐focused approaches, illustrating situated learning theory’s concept of learning the “actual” rather than “intended” curriculum [13].

Technology‐enhanced educational interventions offer partial mitigation: simulation training in controlled, low‐stakes environments allows students to practice complete documentation of the nursing process without clinical time pressures, building systematic habits before deployment in high‐acuity settings [16]. Virtual simulation and standardized patient exercises can efficiently consolidate nursing process competencies that would otherwise require extended, unsupported clinical time. Year 4 students’ significantly fewer reported barriers relative to year 2 students (*p* = 0.027) may reflect improved time management gained through accumulated experience, though it may also partially reflect normalization of incomplete implementation. Systemic solutions—including increased nurse staffing, renegotiated student‐to‐instructor ratios, and negotiated protected learning time—remain essential alongside educational innovations.

### 4.6. Limited Role Modeling and Mentorship: Supplementing With Technology

Nearly one‐third of students (28.3%) rarely observed role models consistently using the nursing process, and 25.5% reported insufficient mentors. This aligns with international evidence documenting inconsistent use of the nursing process in resource‐constrained settings, where task completion takes precedence over systematic care planning [9, 10]. From a social learning perspective, students develop their professional identity through observing experienced practitioners [25]. When preceptors and staff nurses do not visibly model the nursing process, students may perceive it as a theoretical exercise rather than a practical clinical framework.

Flipped learning and technology‐enhanced approaches offer important supplementary pathways: curated nursing process demonstration videos, virtual simulation cases, and structured clinical debriefing sessions can compensate partially for limited in‐person role modeling, providing students with high‐quality exemplars of nursing process application that they may not observe consistently in busy clinical environments [20]. Complementary investments in structured preceptor training programs, appropriate mentor‐to‐student ratios, recognition for teaching roles, and selective placement in clinical sites with strong nursing process cultures remain essential.

### 4.7. Institutional Support and Academic–Practice Partnerships

One‐quarter of students (26.4%) perceived inadequate institutional support from hospitals, and 25.5% felt limited support from medical staff. This suggests that some clinical environments do not prioritize nursing students’ learning or the systematic implementation of the nursing process—likely reflecting hierarchical professional relationships and task‐oriented care cultures prevalent in some Cambodian healthcare settings [15]. Formal academic–practice partnerships with clear learning objectives, designated clinical educators, structured orientation programs, and regular faculty–facility governance communication are needed to build genuinely supportive clinical learning environments.

### 4.8. Integrated Recommendations for Nursing Education: Toward Curriculum Transformation

To enhance clarity and practical application, the recommendations are organized across four key domains: curriculum design, clinical training, learning resources, and system‐level strategies.

#### 4.8.1. Curriculum Design and Pedagogy

• Integrate flipped classroom models into nursing process instruction. Preclass asynchronous activities—such as recorded lectures, case vignettes, and interactive quizzes—can free contact time for simulation, role‐play, and supervised clinical reasoning, thereby maximizing the use of limited educational resources [20].
• Embed FCC principles into nursing process courses using culturally adapted Cambodian patient and family scenarios to strengthen holistic assessment, family engagement, and culturally sensitive care planning [21].

#### 4.8.2. Clinical Training and Supervision

• Expand the use of technology‐enhanced simulation to provide controlled opportunities for students to practice the complete nursing process prior to clinical placement [16].
• Implement structured preceptorship programs with clearly defined roles, expectations, and institutional recognition to strengthen mentorship and role modeling.

#### 4.8.3. Learning Resources and Educational Support

• Develop accessible Khmer‐language resources, including nursing diagnosis handbooks, assessment guides, and care plan templates, to support students’ point‐of‐care learning and application.

#### 4.8.4. System‐Level and Institutional Strategies

• Promote the development of standardized national nursing process documentation through collaboration among the Ministry of Health, educational institutions, and the Cambodian Nurses Association.
• Establish formal academic–practice partnerships between nursing schools and clinical facilities, with shared learning objectives and governance structures to ensure alignment between education and practice.

Although these strategies were not directly evaluated in this study, they are supported by existing evidence and offer practical pathways to address the identified gaps.

### 4.9. Comparison With International Literature and Broader Educational Contribution

The pattern of favorable perceptions coexisting with implementation challenges is consistent across diverse international settings—Ethiopia, Kenya, Pakistan, Iraq, Turkey, Namibia, and Japan [8–12, 19, 22]. Critically, flipped classroom interventions have demonstrated effectiveness in bridging theory–practice gaps in nursing education across multiple international settings [20], and FCC integration has improved holistic care competencies in contexts comparable to Cambodia [21]—providing internationally validated pedagogical models that can be contextualized for the Cambodian setting.

What distinguishes the Cambodian context is a particular constellation of challenges: postconflict health system development, rapid expansion of nursing education with variable quality standards, cultural factors affecting patient–provider communication, the absence of standardized documentation, severe nursing shortages, and limited educational infrastructure. This study adds quantitative evidence to Cambodia’s nascent nursing research literature, extending beyond prior primarily qualitative and practitioner‐focused Cambodian work [14, 15] and contributing to the broader evidence base on educational strategies for LMICs.

#### 4.9.1. Strengths and Limitations

Methodological strengths include a rigorous instrument development process with strong validity (S‐CVI/Ave = 0.93) and good‐to‐excellent reliability (α = 0.82–0.88), an adequate sample size with a good response rate (71.7%), the use of both descriptive and inferential statistics, and grounding in situated learning theory. Limitations include convenience sampling from a single private university, vulnerability of self‐report data to social desirability and recall bias, a cross‐sectional design that precludes causal inference, a limited qualitative component, and the absence of clinical instructor or preceptor perspectives. Unmeasured confounders, including prior healthcare experience and academic performance, may also influence findings.

#### 4.9.2. Future Research Directions

Multisite studies encompassing public universities, provincial institutions, and diploma programs are needed for representative national data. Longitudinal research tracking cohorts from program entry through early professional practice would clarify the trajectories of competency development. Intervention studies rigorously evaluating the effectiveness of flipped classroom implementation, standardized simulation programs, and FCC‐oriented curricula on nursing process competence and patient outcomes are particularly warranted. Mixed‐methods designs, comparative studies across facility types, and stakeholder perspectives from clinical instructors, preceptors, and administrators should also be pursued.

## 5. Conclusion

Nursing students at this private university in Phnom Penh demonstrated positive attitudes toward the nursing process and recognized its importance for organizing care and supporting clinical decision‐making. However, they experienced moderate practical challenges—particularly related to patient assessment, documentation variability, and knowledge application—and meaningful organizational barriers, including heavy workload, limited role modeling, and insufficient institutional support. These findings indicate a theory–practice gap in which positive perceptions do not necessarily translate into consistent clinical application.

The study suggests the need to strengthen alignment between classroom teaching and clinical practice, particularly through improved supervision, mentorship, and more consistent clinical learning environments. Enhancing collaboration between educational institutions and clinical facilities may further support students’ application of the nursing process.

Future curriculum development may consider educational approaches such as simulation‐based learning, flipped classroom strategies, technology‐supported learning, and FCC, as these have been highlighted in the literature as potentially beneficial; however, these approaches were not evaluated in the present study.

Further multisite research involving both public and provincial institutions is recommended to strengthen generalizability and inform evidence‐based educational reform in Cambodia.

## Author Contributions

Sokha Yem: conceptualization, methodology, formal analysis, writing–original draft, writing–review and editing, and project administration. Lida Vann: methodology, data collection, and writing–review and editing. Leapchakriya Loem: data collection, formal analysis, and writing–review and editing. Sokunthea Kem: data collection and writing–review and editing. Sreypeov Tun: data collection and writing–review and editing. Nov Sreyroth: literature review and writing–review and editing.

## Funding

This research received no specific grant from any funding agency in the public, commercial, or not‐for‐profit sectors.

## Disclosure

All authors have read and approved the final manuscript.

## Conflicts of Interest

The authors declare no conflicts of interest.

## Supporting Information

Additional supporting information can be found online in the Supporting Information section.

## Supporting information


**Supporting Information** Table S1. Items revised based on expert feedback during content validity assessment. Table S1 presents the revision process of questionnaire items based on expert evaluation during the content validity assessment. It includes the original items, item‐level content validity index (I‐CVI) results, expert feedback, and the final revised items. Items with I‐CVI below the acceptable threshold (0.67) for relevance, clarity, or simplicity were revised to improve content validity and ensure suitability for student respondents.

## Data Availability

The datasets generated and analyzed during the current study are available from the corresponding author on reasonable request.
