# Supplementary material for: Nursing Students’ Perceptions, Challenges, and Barriers in Applying the Nursing Process During Clinical Training in Cambodia: A Cross‐Sectional Study
Source: Nurs Res Pract. 2026 May 30;2026:6401733. doi: 10.1155/nrp/6401733 (PMC13239052; doi:10.1155/nrp/6401733)
Supplement: Supplementary file 1 — Supporting Information Table S1. Items revised based on expert feedback during content validity assessment. Table S1 presents the revision process of questionnaire items based on expert evaluation during the content validity assessment. It includes the original items, item‐level content validity index (I‐CVI) results, expert feedback, and the final revised items. Items with I‐CVI below the acceptable threshold (0.67) for relevance, clarity, or simplicity were revised to improve content validity and ensure suitability for student respondents. [file NRP-2026-6401733-s001.docx]

**Table S1. Items Revised Based on Expert Feedback During Content Validity Assessment**

| **Item Domain** | **Original Item** | **I-CVI Issue** | **Expert Feedback Summary** | **Revised Item** |
| --- | --- | --- | --- | --- |
| Perceptions | I have adequate knowledge about all steps of the nursing process | Relevance: 0.67; Clarity: 0.33 | Wording too absolute; "adequate knowledge" unclear; does not distinguish understanding from confidence | I understand each step of the nursing process clearly |
| Challenges | Documentation requirements vary significantly between clinical sites | Clarity: 0.67; Simplicity: 0.67 | Too formal for student audience; "requirements" ambiguous; needs more specific focus | Different hospitals use different nursing process forms |
| Challenges | Patients' cognitive or physical limitations impede comprehensive assessment | Simplicity: 0.33; Clarity: 0.67 | Medical jargon inappropriate for students; too technical; needs plain language | Some patients cannot give enough information for a full assessment |
| Barriers | Clinical learning environments lack structured mentorship opportunities | Simplicity: 0.67; Clarity: 0.67 | Too abstract; "structured mentorship opportunities" unclear to students; needs concrete language | There are not enough instructors or mentors to guide me |
| Barriers | Organizational culture does not prioritize systematic nursing care planning | Relevance: 0.67; Simplicity: 0.33 | Too abstract; "organizational culture" beyond student experience; needs institutional focus | Hospitals do not provide enough support or encouragement for nursing care planning |

***Note: I-CVI = Item-level Content Validity Index; acceptable threshold = 0.67 for a three-expert panel (Polit & Beck, 2006). Items with I-CVI < 0.67 on any dimension (relevance, clarity, simplicity) were revised to improve content validity.***
